# Psychosis: The Utility of Ketamine as a Pharmacological Model of Psychotic-like Symptoms in Rodents: A Review of Dosage Regimens

**DOI:** 10.3390/biology15030222

**Published:** 2026-01-25

**Authors:** Claire A. Rice, Robert W. Stackman

**Affiliations:** Department of Psychology, and Stiles-Nicholson Brain Institute, Florida Atlantic University, Jupiter, FL 33458, USA; ricec@health.fau.edu

**Keywords:** schizophrenia, bipolar disorder, ADHD, learning, memory, dosing schedule

## Abstract

Ketamine (KET) administration protocols vary widely in their design, with acute, sub-chronic, and chronic dosing regimens used to induce psychotic-like behavior in rodent models. This review compares representative classic and contemporary studies employing differing KET administration protocols to model psychosis in laboratory rodents. Specifically, we have focused on the behavioral tasks and analytical methods used to validate KET-induced symptoms of psychosis-like and schizophrenia-like behaviors. While variability in behavioral tasks complicates direct comparisons across studies, these findings provide a framework for selecting dosing strategies aligned with specific research objectives. Acute KET protocols are particularly suited for addiction research or as a preliminary approach preceding longer-term studies. In contrast, protocols utilizing repeated or sub-chronic, or chronic administration of KET tend to yield more comprehensive models of psychosis-like behavior and are better suited for examining the associated enduring cognitive and neurobiological impairments. Administering KET intravenously or intraperitoneally at frequent intervals or with a bolus dose, may sustain higher levels of bioavailable KET, thereby producing a more robust and reliable psychosis-like phenotype, especially relevant for investigations of long-term cognitive and neurological dysfunction.

## Simple Summary

Psychotic illnesses such as schizophrenia and bipolar disorder can cause hallucinations, loss of motivation, and serious problems with attention and memory, yet their brain basis is difficult to study directly in people. Researchers therefore use ketamine, an anesthetic and pain medication that, at lower doses, disrupts a major brain signaling system, to produce temporary psychosis-like changes in rodents and to test potential treatments. In this review, we compared published rodent studies that used different ketamine dosing schedules and summarized their impact on behavior test results and measurements of brain chemistry and activity. Across studies, a single ketamine dose reliably caused short-lived overactivity and often reduced attention, but it rarely produced lasting memory or mood changes. In contrast, repeated dosing, especially when followed by a drug-free recovery period, more consistently produced enduring deficits in working and long-term memory, attention, and anxiety- or depression-like behaviors, along with brain changes that resemble those reported in psychotic disorders. Overall, our synthesis provides practical guidance for selecting ketamine regimens matched to specific research goals, helping accelerate the development of effective treatments that reduce disability and the societal burden of psychotic illness.

## 1. Introduction

Psychotic disorders affect over 10 million individuals, approximately 3% of the U.S. population [1], and globally, an estimated 47 million people live with bipolar disorder and 20 million with schizophrenia [2,3]. Dysfunctional synchronization within neural circuits among several brain regions has been implicated in the manifestation of psychotic symptoms [4,5,6]. Studies have identified profound impairments within circuits of the hippocampal formation in individuals with schizophrenia [7]. The hippocampus in schizophrenia and bipolar disorder exhibits abnormal hypermetabolism [7,8], structural atrophy, [9,10,11], and reduced dendritic spine density [12,13]. These hippocampal abnormalities are associated with symptoms such as mania, memory impairment, attention deficits [7], and impaired latent inhibition (LI) [14,15,16]. Additional dysfunction has been well documented in the prefrontal cortex (PFC) [17,18,19,20,21] and the nucleus accumbens [22,23].

To investigate potential treatments for psychotic disorders, various rodent models have been developed, including genetic, neurodevelopmental, lesion-based, and pharmacological models. Notably, pharmacological models offer a cost-effective, time-efficient, and technically straightforward approach to inducing psychosis-like symptoms in rodents, making them particularly attractive for translational research [24,25].

Among pharmacological agents, KET, a non-competitive NMDA receptor antagonist has emerged as a widely used compound to induce psychotic-like behavior in rodent models [26,27,28]. KET administration has been demonstrated to evoke positive- and negative-like symptoms of psychosis [29,30,31] and its ability to model cognitive symptoms associated with psychotic disorders significantly enhances its utility for research. Depending on the dosage regimen, systemic KET administration in rodents can lead to a wide array of behavioral phenotypes, including manic-like behavior, increased anxiety and depression, attentional deficits, and impairments in working, spatial, and long-term memory, mirroring the symptomatic profile typically observed in human patients with psychosis [32].

### 1.1. Symptoms of Psychosis

Schizophrenia is characterized by a constellation of positive, negative, and cognitive symptoms [29,30,31]. Positive symptoms, those that represent the addition of abnormal behaviors, include hallucinations, delusions, formal thought disorder, and bizarre behavior [31,33,34]. In contrast, negative symptoms reflect the loss or diminution of normal function, such as avolition, apathy, alogia, and anhedonia [31,35]. Cognitive symptoms are also central to psychotic disorders and involve disorganized thinking, memory impairments, attentional deficits, and difficulty maintaining focus [32]. Notably, some of these cognitive symptoms overlap with those seen in attention-deficit/hyperactivity disorder (ADHD), which is often comorbid with psychosis [30]. Among these, deficits in attention are particularly debilitating, as they disrupt the ability to complete tasks and to maintain coherent cognitive engagement or verbal expression.

Selective and sustained attention deficits are hallmarks of schizophrenia, bipolar disorder, and ADHD [27,29]. The American Psychiatric Association [36] identifies impaired sustained attention, impulsiveness, and hyperactivity as the core triad of ADHD. In bipolar disorder, distractibility during manic episodes is so prominent that it is included as a diagnostic criterion in the DSM-V [36]. To explain the diverse symptomology observed in psychosis, two major neurobiological frameworks have emerged as follows: the dopamine hypothesis and the glutamate hypothesis. Recent research increasingly supports a convergent model, integrating these mechanisms and offering new insights into the pathophysiology of psychotic disorders.

### 1.2. Hypothesis of Psychosis

#### 1.2.1. The Dopamine Hypothesis

The dopamine hypothesis posits that dysregulated dopaminergic neurotransmission contributes to the development of psychotic symptoms. Dopaminergic agonists such as amphetamines and cocaine elicit schizophrenia-like behaviors by inducing hyperdopaminergia, primarily by inhibiting vesicular monoamine transporter 2 and the dopamine transporter, leading to elevated synaptic dopamine levels [32,37]. These drug-induced changes can result in psychotic symptoms that closely resemble those seen in schizophrenia.

The primary and initial support for a hyperfunctioning dopaminergic system as central to schizophrenia came from evidence that the degree to which antipsychotic medications block dopamine receptors or enhance dopamine reuptake is directly related to their effectiveness in treating psychosis. These agents, particularly D2 receptor antagonists, are therapeutically beneficial in reducing the positive symptoms of schizophrenia, such as hallucinations and delusions [38]. These findings have led to the more focused hypothesis that hyperdopaminergic stimulation of striatal D2 receptors, or their super-sensitivity to dopamine, contributes significantly to the expression of positive symptoms [39,40]. Indeed, results of neuroimaging studies of schizophrenia patients indicate striatal overexpression of D2 receptors [41]. However, both first- and second-generation antipsychotic treatments offer limited efficacy for the negative and cognitive symptoms associated with schizophrenia [42,43,44,45], highlighting a major limitation of the dopamine-centered model, and the need for more focused preclinical research into the neurobiological mechanisms of the cognitive symptoms of schizophrenia.

The dopamine hypothesis has also been extended to bipolar disorder, especially in explaining manic episodes. Amphetamine-induced mania and the efficacy of anti-dopaminergic drugs in treating mania support a role for excess DA activity. Ashok et al. [46] demonstrated both pharmacological and imaging evidence that hyperdopaminergic activity contributes to mania, including heightened activity in the reward circuit and elevated D2 and D3 receptor availability [46]. In contrast, depressive episodes may involve hypodopaminergic states, suggesting that bipolar disorder may involve dynamic shifts in dopamine levels. A proposed mechanism involves impaired homeostatic regulation, wherein the system oscillates between hyper- and hypodopaminergic states as compensatory responses [46]. Despite its strengths, the dopamine hypothesis does not fully account for the spectrum of symptoms characterizing schizophrenia or bipolar disorder, particularly negative and cognitive impairments, prompting the consideration of additional neurochemical systems. Emerging research has therefore focused on the glutamate hypothesis, which may offer a more comprehensive model of psychosis and may also illuminate the neurobiological underpinnings of bipolar disorder.

#### 1.2.2. The Glutamate Hypothesis

While dopaminergic models account for the positive symptoms of schizophrenia, they fall short in addressing the negative and cognitive impairments that are equally debilitating. In contrast, glutamatergic models, particularly those involving NMDA receptor hypofunction, offer a more comprehensive framework, accounting for a broader range of schizophrenia symptoms [47] and extending to the neurobiological underpinnings of bipolar disorder as well [48]. Evidence supporting the glutamate hypothesis emerged from studies showing that NMDA receptor antagonists, such as ketamine (KET) or phencyclidine (PCP), can induce a range of psychotic-like symptoms in humans and laboratory animals [27,49,50]. These drugs not only produced hallucinations and delusions (positive symptoms), but also mimic negative symptoms such as social withdrawal, and cognitive deficits including disorganized thinking and impaired working memory.

The glutamate hypothesis posits that NMDA receptor hypofunction, particularly within cortical and limbic circuits, is a central mechanism contributing to schizophrenia [47,51,52,53,54]. This theory is supported by neurobiological findings from postmortem and imaging studies of individuals with schizophrenia. For example, patients exhibit reduced grey matter volume [54,55,56], decreased NMDA receptor binding [57], and reduced expression of the NR1 subunit of the NMDA receptor [58]. These deficits align with the observed symptomology and lend strong support to the glutamate model. Steeds et al. [47] highlighted that disruptions in glutamatergic signaling may also interact with dopaminergic pathways, suggesting that a dual-deficit model involving both dopamine and glutamate dysfunction that may offer the most accurate neurochemical framework for understanding schizophrenia. In response to these insights, researchers have developed rodent models using NMDA receptor antagonists to induce psychotic-like behaviors. These glutamate-focused models allow for the controlled investigation of cognitive deficits, particularly in memory and attention, and are instrumental in testing novel therapeutics targeting the glutamatergic system.

### 1.3. Rodent Models of Schizophrenia. Bipolar Disorder, and ADHD

Several rodent models have been developed to emulate psychotic-like symptoms using a range of methodological approaches, including genetic, neurodevelopmental, lesion-based, and pharmacological manipulations. These models aim to elucidate the neural mechanisms underlying cognitive dysfunctions commonly associated with schizophrenia, bipolar disorder, and ADHD, particularly deficits in learning and memory, executive function, and attention. Among these, pharmacological models offer a practical and scalable platform for investigating the neurobiology of psychosis. This review focuses specifically on the use of ketamine in rodents as a pharmacological model for inducing psychotic-like symptoms.

### 1.4. Pharmacological Models

#### 1.4.1. The Dopamine Model

Pharmacological rodent models supporting the dopamine hypothesis are partly based in clinical observations that amphetamine-induced psychosis resembles the positive symptoms of schizophrenia. These psychostimulants alter both norepinephrinergic and dopaminergic activity, contributing to behavioral abnormalities [32,59]. Early studies demonstrated that amphetamine administration in rodents results in hyperactivity, and in some cases, behavioral sensitization [60,61]. However, findings have varied, as some report hyperactivity without sensitization [62], and many fail to replicate negative symptoms typically observed in schizophrenia [27,47,63]. These behavioral results are generally consistent with the amphetamine-induced psychosis observed in humans [47,64], although some variability has been noted [65]. Importantly, while some cognitive impairments are evident, particularly on prefrontal cortex-dependent tasks, hippocampal-dependent cognitive functions often remain intact in these models [60,66]. More recent research highlights that the severity and specificity of behavioral and cognitive impairments may be dose-dependent and influenced by the age at which the drug is administered [67]. These findings suggest that while dopaminergic models capture aspects of psychosis, their limitations in modeling the full symptom spectrum, especially negative and cognitive symptoms, reduce their translational utility.

#### 1.4.2. The Glutamate Model

Pharmacological models targeting the glutamate system have gained prominence due to compelling evidence that NMDA receptor hypofunction plays a critical role in the pathophysiology of psychosis. Early work by Konradi & Heckers [53] showed that patients with schizophrenia exhibit dysregulation of the glutamatergic system, particularly reduced NMDA receptor activity. This conclusion was supported by observations that NMDA receptor antagonists, notably KET and PCP, can induce psychosis-like symptoms, including delusions and hallucinations in both humans and laboratory animals [27,50,68]. Jentsch and Roth [49] proposed that PCP could be used to model schizophrenia in rodents, laying the foundation for a range of glutamate-based pharmacological models. PCP-treated mice exhibit hyperlocomotion [69], social withdrawal [63], impaired pre-pulse inhibition [70], and cognitive deficits [71], closely mirroring the symptom clusters observed in human patients.

Glutamate dysfunction is also implicated in bipolar disorder, particularly during manic episodes. Animal models such as amphetamine-induced hyperactivity [72] and ouabain injection [73], which is based on Na+/K+-ATPase dysfunction, have been shown to produce manic-like behaviors. These results are supported by reports of altered glutamate receptor activity [74,75], and reduced expression of NMDA receptor subunits in the hippocampus of bipolar patients [76]. Furthermore, KET has been shown to increase the glutamate release by disinhibiting GABAergic inputs and thereby elevating neuronal firing rates in a manner consistent with manic states [77].

These findings indicate that the glutamate hypothesis does not compete with the dopamine hypothesis; instead it complements and extends it as an integrated explanatory framework. The two systems likely interact dynamically in psychotic disorders. This integrated view is supported by results from biochemical and molecular studies in rodents treated with acute and sub-chronic KET, which have revealed complex, region-specific changes in the prefrontal cortex, hippocampus, and striatum [78]. In turn, Chatterjee et al. [78] proposed a schematic illustrating the multi-symptom profile of KET-induced psychosis. Briefly, their proposal suggested that positive symptoms arise from NMDA receptor blockade on GABAergic neurons, leading to reduced GABA release, and consequently increased dopamine neurotransmission, and D2 receptor hyperfunction in the striatum. Chatterjee and colleagues [78] proposed that negative symptoms result from two mechanisms: (1) disinhibition of 5-HT neurons increasing 5-HT release and 5HT2 receptor hyperfunction in the cortex, or (2) NMDA receptor-mediated glycine inhibition. Finally, cognitive symptoms are proposed to arise from NMDA receptor blockade inhibiting neuronal nitric oxide synthases (nNOS) in the hippocampus and suppression of nicotinic acetylcholine receptor (nAChR) activity, which reduces glutamate release. Together, these mechanisms provide a robust framework for understanding the interconnected roles of glutamate, dopamine, serotonin, and acetylcholine systems in psychosis. KET-induced rodent models thus offer a powerful tool for probing the multifaceted neurobiology of schizophrenia, bipolar disorder, and related cognitive impairments.

### 1.5. Ketamine as a Pharmacological Model of Psychosis

Ketamine (2-chlorphenyl-2-methylamino-cyclohexanone, KET) is a PCP derivative initially developed as a safer anesthetic with analgesic and amnesic properties [79,80]. Beyond its clinical applications, KET has gained recognition in psychiatric research for its ability to model psychotic symptoms, and more recently, as a fast-acting anti-depressant [81,82]. First synthesized from PCP in 1962 to minimize PCP’s neurotoxicity [80], KET has since become widely used in both human and animal studies to induce symptoms relevant to schizophrenia and bipolar disorder.

In human studies, acute administration of KET transiently increases positive (e.g., hallucinations and delusions), negative (e.g., blunting of affect), and cognitive symptoms (e.g., impaired memory and attention) in both individuals with schizophrenia and healthy controls [47,50,83,84,85]. While schizophrenic patients typically experience auditory hallucinations, healthy participants receiving acute KET more often report visual hallucinations [47,86]. Interestingly, chronic PCP users report both auditory and visual hallucinations [49]. However, acute KET models are limited in their ability to simulate long-term changes in brain structure and function associated with chronic psychosis [87,88].

In contrast, sub-chronic (<3 mos) and chronic (>3 mos) KET use in humans has been associated with more persistent schizophrenia-like symptoms, including delusions, cognitive impairments, depression, and disassociation [49,50,64,84,86,89]. Studies have demonstrated that chronic usage of KET or PCP induces chemical and structural changes in brain region connectivity similar to those seen in schizophrenic individuals. These changes were seen in both human studies and animal models [47,90,91,92].

In rodents, chronic KET administration produces prolonged behavioral and cognitive changes consistent with schizophrenia, including impairments in hippocampal memory, working memory, and fear learning [89,93], mirroring those observed in schizophrenic patients [94,95,96]. These deficits in KET-treated rodents are typically assessed using tasks such as the novel object recognition task [89,97,98] and trace fear conditioning protocols [24,99,100,101].

Like KET, PCP administration in animals also induces changes in brain structure that resemble those seen in schizophrenia, including reductions in gray matter volume and alterations in neurotransmitter signaling [27,49]. Functional neuroimaging studies have linked KET’s NMDA receptor antagonism to disruptions in prefrontal and limbic circuits. For instance, the NMDA receptor blockade is associated with negative symptoms [102], while increased glutamate/glutamine levels and altered blood flow patterns in the prefrontal cortex are correlated with positive symptoms [47,77,85,103,104]. Reductions in thalamic n-acetyl aspartate [92], prefrontal grey matter volume [90], and frontal cortex blood flow [84,105] have all been observed in chronic KET or PCP users, mirroring abnormalities in patients with psychosis [55].

Despite its widespread application in preclinical studies of psychosis-like symptomology, KET administration protocols vary widely in terms of dose, route, frequency, and duration. Previous reviews on KET have addressed several topics in KET research including its efficacy for anesthesia, analgesia [106,107], influencing depression [108,109,110,111] and fear memory [112,113], and its effects on the dopaminergic system [114,115]. Further, while there are numerous reports of the efficacy of KET to elicit psychotic- and schizophrenia-like behavioral and neurological symptoms in rodents, the dosing protocols reported vary markedly. Therefore, this review preferentially focuses on comparing acute, sub-chronic, and chronic KET regimens in rodent models to help researchers determine the most appropriate dosing strategy to meet specific experimental goals. While definitions of “sub-chronic” and “chronic” vary across studies, we define sub-chronic administration as treatment lasting ≤30 days. Notably, dose equivalency between mice and rats is approximately 2:1, respectively [116,117]. The review highlights findings from classic and contemporary studies across several behavioral tasks, emphasizing the need to align KET protocols with the cognitive, affective, or neurochemical dimensions of interest.

## 2. Materials and Methods

Following the model described by Nakahara et al. [11], a systematic search of the National Library of Medicine’s PubMed database was conducted. A search using the terms, *ketamine* and *rodent models* yielded nearly 2900 articles; adding the term *neurological disorder* reduced the yield to 620. As our effort here was focused on modeling psychosis-like symptoms, we revised the search using the terms: *ketamine, psychosis, rodents*, and *ketamine, schizophrenia,* and *rodents*. The search yielded 261 results: *KET-induced psychosis in rodents* (*n* = 120), and *KET-induced schizophrenia in rodents* (*n* = 141). After removing duplicates, full-text screening was performed based on inclusion criteria focused on dosing strategy, behavioral assessment, and neurobiological outcomes. This process identified 28 representative peer-reviewed articles suitable for final analysis (see Table 1). Ranging from foundational to recent, this selection of studies was chosen to compare the consequences of KET administration across biochemical, neurochemical, immunohistochemical, electrophysiological, and behavioral determinants. Further, these studies were analyzed to capture a range of dosing regimens, including variations in dose, frequency, treatment duration, washout periods, and species/strain of rodents.

Of these, 12 studies included direct assessments of brain structure, neurochemistry, and/or electrophysiological responses, enabling comparisons to known neurobiological features observed in schizophrenic or bipolar patients.

## 3. Results—Review of Ketamine Protocols

### 3.1. Neurochemical Analysis

#### 3.1.1. Neurochemical Effects of Acute Administration

Ishiyama et al. [141] examined the effects of acute KET administration (12 mg/kg SC) in Wistar rats and observed reductions in glutamate, dopamine, and HIAA/5-HT turnover specifically in the dentate gyrus. While plasma corticosterone levels increased significantly, there were no significant changes in brain tissue concentrations of noradrenaline, 5-HT, or the dopamine metabolite DOPAC, nor in the DOPAC/dopamine ratio [141]. In a related study, Razoux et al. [120] reported that acute KET (25 mg/kg, IP) significantly elevated extracellular glutamate levels in the nucleus accumbens shell of Wister rats, as measured by in vivo microdialysis. This elevated glutamate response is consistent with earlier findings demonstrating KET-induced increases in both glutamate and dopamine efflux in the nucleus accumbens [77,142].

These neurochemical changes align with postmortem findings in schizophrenia patients, which include elevated glutamate/glutamine ratios, as well as reduced expression of NMDA receptor NR2 subunits [143,144,145]. Similar alterations have also been reported in bipolar patients, particularly during manic episodes, as reflected by increased serum glutamate concentrations [146].

#### 3.1.2. Neurochemical Effects of Sub-Chronic Administration

Sub-chronic KET treatment in rodent models reveals distinct neurochemical and oxidative changes that may underlie the pathophysiology of psychotic and mood disorders. These effects often exhibit brain-region and sex-specific patterns. Thelen et al. [140] reported sex-dependent alterations in hippocampal neurochemistry after administration of 10 mg/kg KET IP, once daily for 21 days, followed by a 24 h washout: female C57BL/6J mice had reduced glutamate and aspartate levels, while male mice had elevated 5-HIAA and 5-HIAA/5-HT ratios, suggesting increased 5-HT turnover. No significant changes were found in the prefrontal cortex. In males, repeated KET treatment also increased synapsin-I protein expression and SNARE 100 kDa complexes in the hippocampus, consistent with an anti-depressant-like phenotype [140].

Ghedim et al. [124] administered 25 mg/kg KET IP to Wistar rats once daily for 14 days, followed by an acute dose on day 15, and found increased thiobarbituric acid-reactive substances (TBARS), a consequence of lipid peroxidation in the prefrontal cortex, hippocampus, striatum, and amygdala. These increases parallel elevated serum TBARS levels observed in patients during manic or late-stage bipolar episodes [88,147,148,149]. They also reported protein carbonylation in several brain regions, mimicking the oxidative protein damage found in bipolar disorder [150,151,152].

Gazal et al. [122] confirmed these results using a similar sub-chronic KET regimen (25 mg/kg IP daily for 7 days, with an acute dose on day 8) and reported decreased thiol content in the prefrontal cortex and reduced superoxide dismutase (SOD) and catalase activity in the prefrontal cortex and hippocampus, further implicating disruptions in antioxidant systems in KET-induced oxidative stress.

In contrast, Arslan et al. [123] used the same KET dose and treatment duration in Sprague–Dawley rats, followed by a 30 min washout, and found no significant changes in TBARS, catalase, or SOD activity in the prefrontal cortex or hippocampus. While these results are not consistent with prior findings, they may suggest that oxidative stress markers vary by disease phase, often increasing during acute episodes but normalizing during euthymia [147] or late stages [153].

Hou et al. [136] explored dose-dependent effects by treating Swiss–Kunming mice with 25, 50, or 100 mg/kg KET IP for 7 days, followed by an acute dose on day 8 and no washout. Results showed dose-dependent decreases in SOD levels in both serum and hippocampus, as well as elevated nitric oxide and total nitric oxide synthase (TNOS) in the serum, prefrontal cortex, and hippocampus. These results suggest that sub-chronic KET exposure induces oxidative stress and may contribute to neurotoxic injury [136].

### 3.2. Immunohistochemistry

#### 3.2.1. Immunohistochemical Analyses After Acute Administration

Immunohistochemical analysis following acute KET administration reveals region-specific changes in immediate early gene expression. Imre et al. [118] administered KET at 4, 8, 12, or 16 mg/kg SC to Wistar rats and observed dose-dependent increases in c-Fos-positive cells in several brain regions, including the prefrontal cortex, retrosplenial cortex, hippocampus, nucleus accumbens, amygdala, and hypothalamus. These findings align with earlier results by Duncan et al. [154], who also reported increased c-Fos expression following acute KET. Interestingly, lower doses (4 mg/kg) resulted in elevated c-Fos expression in the CA1, CA3, and dentate gyrus regions of the hippocampus, as well as in the central nucleus of the amygdala. However, higher doses resulted in attenuated c-Fos expression in these same regions, suggesting a non-linear, dose-dependent response. These findings imply that acute KET administration induces widespread neuronal activation, but that higher doses may suppress activity in key limbic regions, potentially modeling dissociative or negative symptoms of psychosis.

#### 3.2.2. Immunohistochemical Analyses After Sub-Chronic Administration

Sub-chronic KET exposure appears to reduce neuronal activation and disrupt interneuron function in regions critical for memory and other cognitive functions. Hauser et al. [97] treated C57BL6N mice with 30 mg/kg KET IP once daily for 14 days, followed by a 24 h washout, and reported a significant decrease in c-Fos-positive neurons in both temporal CA1 and septal subiculum. Moreover, there was a reduction in parvalbumin-expressing interneurons in both the medial prefrontal cortex and dorsal hippocampus, consistent with previous studies [155,156,157]. Sub-chronic 30 mg/kg treatment of male and female Wistar rats for 5 days reduced prefrontal cortical expression of mRNA for the neurotrophin BDNF [133]. These results are notable because parvalbumin-positive interneurons play a critical role in regulating cortical oscillations and cognitive function, processes often disrupted in schizophrenia. The reduced expression of c-Fos and parvalbumin following sub-chronic KET administration correlates with observed memory impairments in behavioral tasks and provides a reliable neurobiological marker for the induction of psychosis-like states in rodent models.

### 3.3. Electrophysiological Effects of Acute and Sub-Chronic Administration

Electrophysiological studies provide critical insight into the acute effects of KET on excitatory neurotransmission within circuits implicated in psychotic-like behavior. Razoux et al. [120] administered an acute IP dose of 25 mg/kg KET to Wistar rats and observed a transient increase in the amplitude of prefrontal cortex-evoked excitatory postsynaptic potentials (EPSPs) in the shell region of the nucleus accumbens. This increase was observed between 10 and 40 min post-injection and coincided with the onset of hyperlocomotion, stereotypic behavior, and ataxia—features of the KET “alpha phase”. These results support the model proposed by Takahata & Moghaddam [158], which suggests that the behavioral effects of NMDA antagonists like PCP and KET are mediated via disrupted glutamatergic projections from the prefrontal cortex to subcortical structures, including the nucleus accumbens and ventral tegmental area. Concomitantly, reduced glutamatergic signaling from the hippocampus to nucleus accumbens may further contribute to this dysregulation [158,159].

KET and PCP bind within the ion channel pore of NMDA receptors, a site accessible under depolarized conditions when the Mg^2+^ block is relieved. Consequently, KET preferentially impacts NMDA receptors on tonic firing GABAergic interneurons over burst firing pyramidal neurons due to their sustained depolarization [160,161,162]. This leads to disinhibition of excitatory pyramidal neurons within cortical and limbic circuits, potentially underlying KET-induced behavioral phenotypes that resemble psychosis. These disruptions in cortico–basal ganglia signaling are hypothesized to underlie core symptom domains of schizophrenia, including impaired salience attribution, disorganized thinking, and aberrant motivation [163].

An early report [128] revealed that systemic administration of KET (80 mg/kg IP), repeated daily, induced high-amplitude slow-wave bursting and spontaneous seizure-like activity in EEG recordings from the amygdala and hippocampus of freely moving Sprague–Dawley rats. A similar pattern of results were found after daily 30 mg/kg administration that was repeated for months [128]. A more comprehensive view of KET’s neurophysiological impact has emerged through system-level analyses using techniques such as local field potential (LFP) and multi-structure microelectrode array recordings. In anesthetized rats, sub-anesthetic doses of KET reduced firing rates in reticular thalamic and prefrontal cortical neurons, decreased delta (1–4 Hz) power, and elevated beta (12–30 Hz) and gamma (30–100 Hz) power [130]. In contrast, freely moving rats given 50 mg/kg KET IP showed increased firing rates in the reuniens thalamus and CA1 region of hippocampus, along with elevated delta and gamma power in both hippocampus and thalamus [131]. These effects of KET mirror EEG abnormalities, particularly increased delta and gamma oscillations, commonly observed in schizophrenia patients [164].

Nasretdinov et al. [132] utilized chronic, large-scale electrode arrays in Sprague–Dawley rats to assess neural dynamics across prefrontal cortex, striatal, basal ganglia, thalamic, and limbic regions. Following acute KET administration (25 or 50 mg/kg IP), they observed significant increases in average LFP power across these structures, coupled with a pronounced reduction in functional connectivity, measured as correlated activity between electrode pairs, both within and between brain regions. These findings suggest that while KET enhances localized neuronal activity, it simultaneously disrupts network-level coherence across cortico–basal ganglia–thalamic circuits. Such desynchronization may contribute to cognitive and sensorimotor deficits characteristic of psychotic disorders.

### 3.4. Behavioral Tasks to Measure Psychotic-like Symptoms

A broad array of behavioral tasks has been employed to validate KET-induced rodent models of schizophrenia and bipolar disorder. Although rodents cannot perform standardized cognitive batteries employed in human studies, many behavioral tasks have strong ecological validity and can be effectively modified for preclinical models. As Powell and Miyakawa [28] noted, one symptom commonly observed in schizophrenia is “psychomotor agitation”, which manifests in rodents as hyperactivity and stereotypic movements. The open field task is widely used to measure changes in locomotor activity and is among the most frequently employed tasks to establish construct validity in KET-based rodent models of psychosis. Thirteen of the 21 reviewed studies used the open field task and consistently reported increased locomotor activity following both acute and sub-chronic KET administration (see Table 1).

Beyond locomotion, sixteen studies employed additional tasks to assess cognitive impairments, particularly those involving hippocampal and prefrontal cortex function, brain regions known to be disrupted in schizophrenia and bipolar disorder. For example, novel object recognition and trace fear conditioning tasks are frequently used to assess long-term hippocampal memory in rodents and both have demonstrated cross-species validity [28,165,166,167,168,169,170]. Tasks such as the association mismatch task [171] assess spatial memory, while working memory, one of the most reproducible cognitive deficits in schizophrenia [172,173], is examined using tasks like the Y-maze, radial-arm maze, and the delay match-to-sample (DMTS) tests. The discrete paired-trial variable-delay T-maze task closely mirrors human delayed non-match to sample tasks and is predictive of prefrontal cortex-related working memory deficits [174].

Sensorimotor gating, another domain disrupted in psychosis, is often tested using pre-pulse inhibition of the startle reflex, a reliable correlate of human deficits [175,176]. Impaired latent inhibition, another hallmark of schizophrenia patients [177,178], is well modeled using established rodent protocols [179,180].

To assess attention and executive function, studies frequently employ the five-choice serial reaction time task (5-CSRTT), adapted from sustained attention tasks in humans [181]. Similarly, the attentional set-shifting task parallels the Wisconsin Card Sorting Test, measuring cognitive flexibility [182,183]. Trace fear conditioning protocols can also be used to measure attention in rodents [184].

Negative symptoms such as anhedonia, behavioral despair, and deficits in social interaction are assessed in rodents using tasks like thermal nociception [185], forced swim test [186], sucrose preference [187], elevated plus maze [188], neophagia [103], and tests of social interaction and social memory.

Together, these behavioral protocols offer robust and diverse metrics for evaluating KET-induced psychosis-like states in rodents. While this list is not exhaustive, it reflects the core behavioral assays used across the 28 representative studies reviewed here and highlights the translational relevance of rodent behavioral phenotyping in preclinical psychiatric research.

### 3.5. Ketamine Dosing Regimens

A total of 28 rodent studies were analyzed to assess the behavioral and neurobiological effects of differing KET dosing regimens. Thirteen studies used rats, with acute doses ranging from 4 mg/kg subcutaneous to 25 mg/kg IP, and sub-chronic schedules ranging from 25 mg/kg to 80 mg/kg IP once daily for up to three months. One study employed intravenous administration with washout periods of 0 to 60 min. Rat strains included Lister, Wistar, Sprague–Dawley, and Long–Evans.

Eight studies used mice, with acute doses ranging from 5 mg/kg to 100 mg/kg IP; and sub-chronic regimens from 4 mg/kg to 100 mg/kg, administered over 5 to 21 days with washout periods of 0 min to 14 days. Mouse strains included C57BL/6J, 129SvPasIc, C57BL/6JOlaHsd, C57Bl6N, and Swiss–Kunming (see Table 1). Dose equivalents between mice and rats are generally estimated at a 2:1 ratio, respectively [116,117].

#### 3.5.1. Acute KET Administration: Locomotor Activity

Across all acute dosing studies, hyperlocomotor activity was reliably observed in the open field assay, regardless of species, strain, or dose. This included doses as low as 4 mg/kg SC, in Wistar rats [119]. Similarly, sub-chronic KET regimens also induced hyperlocomotion when followed by an acute challenge dose, with behavioral effects observed in both rat and mouse models. For example: 25 mg/kg IP with no washout period in Wistar rats [122]; 25 mg/kg IP with a 30 min washout in Sprague–Dawley rats [123,124]; or 20 mg/kg IP with a 1 h washout in C57BL/J6 mice [137]. It is noteworthy that both racemic Ket and S-KET were found to induce a similar profile of significantly increased locomotor activity in rats [121]. These findings confirm hyperactivity as a robust, cross-protocol behavioral response to KET.

#### 3.5.2. Acute KET Administration: Attention

Deficits in attention were frequently reported after acute KET administration, even at low doses: impaired pre-pulse inhibition after 4 mg/kg SC and impaired performance in the discrete paired-trial delayed T-maze task after 12 mg/kg SC in Wistar rats [118,119]; pre-pulse inhibition was disrupted at higher doses of 100 mg/kg IP in C57BL/6J and 129SvPasIco mice [134]; impaired sustained attention was measured in the 5-CSRTT after 10 mg/kg IP KET in C57BL/6JOlaHsd and CD1 mice [135]; impaired latent inhibition in an eyeblink conditioning task in Wistar rats after a 25 mg/kg IP dose [120]; and impaired attentional set shifting after 20 mg/kg IP to C57BL/6J mice [137]. These findings underscore that acute KET administration can elicit significant attentional deficits at rather low doses, whereas equivalent sub-chronic regimens may fail to replicate these effects, highlighting important dose- and duration-dependent differences in KET’s cognitive impact.

#### 3.5.3. Acute KET Administration: Working Memory

Acute KET administration has been shown to impair working memory even at low doses. For example, Wistar rats exhibited significant deficits in the discrete paired-trial variable-delay T-maze task after a 12 mg/kg IP dose [118]. At the other end of the dosing spectrum, Swiss–Kunming mice exhibited working memory impairments in the Y-maze following a 100 mg/kg IP dose [136]. These results support the conclusion that acute KET administration, at both low and high doses, can reliably impair working memory. However, strain-specific differences may moderate these effects and warrant further investigation to optimize dosing protocols.

#### 3.5.4. Sub-Chronic KET Administration: Attention

Sub-chronic dosing regimens have consistently demonstrated attention deficits in rodents, even at modest doses: in Sprague–Dawley rats, 30 mg/kg KET IP once daily for 10 days (but not 5), with a 14-day washout, impaired performance on the attentional set-shifting task [126]; and in male C57BL/6J mice, 30 mg/kg IP, twice daily for 7 days followed by a 7-day washout, impaired attention in a latent inhibition conditioning protocol [189]. These results suggest that both that the total dose and inclusion of a washout period (>5 days) are critical to revealing attention deficits following sub-chronic KET exposure.

#### 3.5.5. Sub-Chronic KET Administration: Working Memory

Sub-chronic KET administration also impairs working memory in a range of behavioral tasks: in Lister rats, 10 and 30 mg/kg IP, once daily for 5 days followed by a 2-day washout, caused persistent impairment (>21 days) in the NMTS/OST task [125]; in Swiss–Kunming mice, 25–100 mg/kg IP KET, once daily for 7 days with no washout but followed by an acute challenge dose, impaired Y-maze performance [136]; in C57BL/6J mice 20 mg/kg IP, once daily for 7 days, with a 7-day washout, impaired attentional set-shifting [137]; and in Long–Evans rats, 30 mg/kg IP, twice daily for 5–7 days with a 10-day washout, impaired performance on both a prefrontal cortex-dependent delayed spatial win-shift maze task [127] and in a hippocampal-dependent delayed match-to-place T-maze task [93], but had no effect on the rats’ performance in a random foraging task [127].

Taken together, these findings show that sub-chronic KET impairs working memory across tasks and species, with long-lasting effects even at moderate doses, especially when a washout period is included or a challenge dose is administered prior to behavioral testing.

#### 3.5.6. Sub-Chronic KET Administration: Long-Term Memory

Long-term memory impairments have been demonstrated following sub-chronic KET exposure: 30 mg/kg IP, once daily for 5 or 14 days with a 24 h washout impaired object recognition memory in Wistar rats and C57BL/6N mice, respectively [97,133]; 100 mg/kg IP, once daily for 7 days with no washout failed to impair object recognition memory in Swiss–Kunming mice [136], demonstrating the importance of post-treatment intervals; and 30 mg/kg IP, once daily for 5 days with a 10-day washout impaired spatial memory in a T-maze working memory task, but enhanced attention in an associative mismatch task, and the detection of a novel object and a novel location in Long–Evans rats [93]. These results indicate that impairment in long-term hippocampal memory can be induced by either lower doses administered over more days with shorter washouts, or by higher doses administered over fewer days with longer washouts. The inclusion of a washout period prior to behavioral testing appears critical to revealing persistent memory deficits.

#### 3.5.7. Sub-Chronic KET Administration: Depression, Anxiety, and Behavioral Despair

Sub-chronic KET administration induces both anti-depressant- and anxiogenic-like effects in rodents, with outcomes strongly influenced by dose, sex, strain, and the presence or absence of a washout period. Thelen et al. [140] administered 10 mg/kg IP KET once daily for 21 days to C57BL/6J mice, followed by a 24 h washout. Sex-specific behavioral differences were observed; specifically, male mice exhibited anti-depressant-like effects, evidenced by reduced immobility in the forced swim test, while female mice displayed increased depressive-like behavior, with greater immobility in the forced swim test. Similarly, 30 mg/kg KET administered to Wistar rats for 5 days reduced immobility (i.e., anti-depressant-like response) in females but had no effect in males [133]. Females also spent significantly less time in the center of the open field arena, indicating heightened anxiety compared to males. Hou et al. [136] found that male Swiss–Kunming mice administered 100 mg/kg KET, once daily for 7 days with no washout period, exhibited increased depressive-like behavior in the forced swim test. Sultana & Lee reported similar increased despair- or depressive-like behavior in C57BL/6J mice after 30 mg/kg IP KET for 5 days [138]. Schumacher et al. [93] reported reduced anxiety in male Long–Evans rats following 30 mg/kg IP KET, twice daily for 7 days, with a 10-day washout. Reduced anxiety was inferred by the reported decrease in time spent in the closed arms of an elevated plus maze, a test sensitive to drugs that are anxiolytic in humans. Reduced social interaction and impaired social recognition memory was also reported after 30 mg/kg IP KET for 5 days in C57BL/6J mice [138], but the same sub-chronic KET treatment failed to elicit these impairments in C57BL/6N mice [139]

Notably, none of the 28 reviewed studies reported significant changes in neophagia, a test commonly used to assess depression-related anhedonia. These findings indicate that sub-chronic KET administration can produce robust behavioral effects associated with depression and anxiety, observable across species and tasks. Furthermore, the data underscore the critical role of biological sex, genetic background strain, and experimental design (e.g., washout duration) in determining behavioral outcomes. Researchers should account for these variables when optimizing dosing regimens and selecting behavioral assays.

## 4. Discussion

The goal of this review was to analyze behavioral and neurobiological consequences of different KET dosing regimens administered to rats and mice, with the objective of guiding researchers in selecting protocols that best match their experimental aims. While acute KET administration is frequently used in addiction models, preclinical studies of anti-depressant action, or to test pharmacological interventions, sub-chronic and chronic regimens more effectively recapitulate a broader range of psychosis-like symptoms, particularly those with long-term cognitive or affective dimensions.

Our comparative analysis of 28 peer-reviewed articles identified a consistent pattern in KET effects that depended primarily on dose and exposure duration. In acute protocols, even low doses reliably increased open field locomotion (often interpreted as a proxy for psychomotor agitation), while mid-to-high doses impaired attention and only very high doses produced working memory deficits. Acute KET did not, however, produce lasting long-term memory impairment or robust anxiety- or depressive-like behavioral changes. Neurochemical findings converged with these behavioral effects, showing glutamatergic alterations and NMDA receptor-related modulation that parallel abnormalities reported in psychotic disorders, supporting the translational relevance of acute KET models. In contrast, sub-chronic and chronic regimens produced outcomes that varied with dose, regimen duration, and washout interval: hyperlocomotion often diminished after washout (suggesting adaptation/tolerance), yet cognitive and affective disturbances commonly persisted, including impairments in attention, working and spatial memory, and long-term memory, alongside increased depression- and anxiety-like markers. Across studies, sub-chronic or chronic KET exposure more consistently generated enduring cognitive and emotional dysfunction and, together with neurochemical and electrophysiological evidence, supported the validity of sub-chronic or chronic KET as a model of psychosis-like pathology.

This review emphasizes the importance of selecting a KET dosing strategy appropriate for the intended experimental objectives. Acute administration of KET produced reliable hyperlocomotion and transient cognitive effects but lacked persistent deficits unless followed by sub-chronic exposure. In contrast, sub-chronic and chronic dosing, especially with appropriately timed washout periods, generated sustained and neurophysiological impairments that more closely mimic schizophrenia and bipolar disorder. Key behavioral findings included (i) working and long-term memory impairments following acute and sub-chronic dosing using object recognition memory tasks [89,97,133,189]; (ii) spatial memory deficits in the delayed T-maze task [93]; (iii) working memory impairments demonstrated across multiple tasks (e.g., Y-maze, radial arm maze, DNMTS) [118,125,127,136]; attention deficits in pre-pulse inhibition, set shifting, latent inhibition, 5-CSRTT, and trace fear conditioning after both acute and sub-chronic KET [118,119,120,126,129,134,135,189]; and (iv) alterations in anxiety-like and depression-like behaviors were induced by both high-dose, short-duration and low-dose, long-duration KET protocols [93,120,136].

### 4.1. The Role of KET Dosing Phases, Washout Timing, and Route of Administration

Behavioral effects of KET depend not only on dose but also on timing. The pharmacodynamic profile includes the half-life phase (5–15 min) characterized by ataxia or cataplexy; the alpha phase (15–40 min) marked by hyperlocomotion and stereotypy [129]; and the beta phase when behavior is returning to baseline or showing sedative-like effects. Studies indicate that the alpha phase aligns with behavioral testing in many acute dosing models and may not fully capture persistent psychosis-like symptoms. Thus, sub-chronic regimens with post-administration washout periods are more effective in modeling enduring cognitive impairments [89,97,189]. Washout periods are especially critical for attention impairments, and both low doses with long washouts and moderate doses followed by short washouts yielded significant deficits [126,137,189]. For working memory, even low dose KET with short washouts (e.g., 2 days) resulted in long-lasting deficits [125]. Anxiety and depression-like symptoms were sensitive to both dose and washout length [93,136,140], reinforcing the need for precise protocol design.

While IP administration is preferred for practicality, analyses of blood serum levels indicate that this route produces peaks and troughs in plasma levels unlike IV infusions, which better mimics human pharmacokinetics [129]. Some studies suggest that more frequently IP dosing (e.g., twice daily) helps sustain bioavailability and enhance translational relevance [129,189,190].

### 4.2. Neurochemical and Structural Validation

The behavioral impairments induced by sub-chronic KET correlated with broad neurobiological changes. For example, (i) elevated levels of glutamate/glutamine and reduced expression of NMDA receptor subunits [118,119,120]; (ii) elevated oxidative stress markers and protein damage in key brain regions, prefrontal cortex, hippocampus, striatum, and amygdala, mirroring findings in bipolar disorder [124,151]; (iii) sex-dependent increases in hippocampal 5-HT turnover and synaptic proteins [140]; and (iv) decreased c-Fos-, BDNF, and parvalbumin-positive interneurons in cortical and hippocampal regions [97,133], consistent with postmortem studies of psychosis patients [155,157].

### 4.3. Behavioral Tasks—Relevance to Disorder Symptomology

While some behavioral tasks for rodents are well suited for translation of results to human behavior, others face limitations due to being based on face or predictive validity. Throughout this review, we have purposely referred to the ability of ketamine to induce behavioral changes in rodents that are *psychotic-like* or *schizophrenia-like* since the altered behaviors in rodents bear some resemblance to the human disordered behavior, but it is not a perfect match. Human task parameters may not be able to be implemented as they are non-existent in rodents, such as guilt, self-confidence or delusions, while others like punishment and probabilities, cannot be matched between species [191]. Hyperlocomotion is used extensively as a measure of manic-like behavior, as the core positive symptoms of schizophrenia; however, hyperlocomotion must also be recognized as nonspecific arousal state that likely interferes with the expression of other high-order behaviors. For example, hyperlocomotion or persistent arousal will complicate the interpretation of reduced social interaction or impaired social or object recognition memory. The challenge is even more concerning when one considers how to interpret ketamine’s effects in a test in which intact memory is inferred from a lack of movement, such as the conditioned freezing response in fear conditioning. Thus, it is critical, where possible, to include measures of higher order behaviors that are less confounded by movement differences.

The forced swim test, which is sensitive to drugs that are effective anti-depressants in humans, has good reliability and predictive validity, but poor construct validity [192]. The common measure of immobility after struggling to escape the forced swim test is considered to reflect despair but could also represent the rapidly learned response to stress, or the shift to a passive coping mechanism. Some have argued that likening immobility to behavioral despair borders on anthropomorphism. Either way, numerous trials of putative anti-depressant drugs that demonstrate promising results in rodents in the forced swim test go on to fail in human trials; thus, there are significant concerns regarding the reliability of the forced swim test as a screen for depressive-like behavior [193,194].

Tests designed to measure anxiety in rodents carry similar interpretational challenges in relating rodent behavior to that of the human feeling of anxiety. The most commonly used tests are those in which administration of drugs that reduce anxiety in humans are also effective in changing approach-avoidance behavioral profiles in rodents [195]. Tasks measuring anxiety-like behavior in rodents are challenged by the need to distinguish fear, the escape response to immediate danger, from anxiety, the response to potential threats or danger [196]. Tests such as novelty-suppressed feeding may potentially confuse anxiety and anhedonia. Often tasks that measure cognition, such as set-shifting and reversal learning, and tasks sensitive to stress and aging, such as open field, are susceptible to non-cognitive factors such as age, anxiety, and motor deficits, that mask true cognitive impairments [197]. These limitations should be considered in the experimental design and interpretation of differing KET dosing schedules. To capture stable not just transient states, the use of multiple tests for the same function should be considered.

### 4.4. Pharmacokinetics

A comprehensive review of KET metabolism and pharmacokinetics [198] revealed the following: KET has low binding to plasma proteins [199] and is widely distributed due to its liposolubility [198]. KET is metabolized to norketamine, an active metabolite, mainly hydroxylized in 6-hydroxy-norketamine and excreted in urine and bile following glucuronoconjugation [198]. The pharmacokinetics are not well understood. Other lesser metabolites are also produced, including 4-OH-norketamine, 6-OH-norketamine, and 5-OH-norketamine [198]. Norketamine is found in blood within 2–3 min and peaks in approximately 30 min, remaining more than 5 h, following a KET IV bolus [200,201]. Sites of metabolism include the liver, kidneys, intestine, and lungs, particularly in animals [202]. The elimination clearance of KET is 1000–1600 mL/min or 12–20 mL/min/kg [203] compared to norketamine [204,205]. The half-life is 2–3 h. Clearance of KET may be 20% high for females than males [206]]. Due to the accumulation of norketamine, less KET is needed over time when IV administered by [204,206]. The combination of the rapid half-life of KET and the lasting presence of its active metabolite may provide support for the contention that twice daily administration of sub-chronic KET provides lasting suppression of NMDA receptor activity which effectively induces persistent cognitive deficits even after washout of KET.

When administered IV, KET reaches its receptors at a high rate with a transfer half-life of less than 1 min, while intramuscular administration has a bioavailability of approximately 93%, with a plasma peak of 5 min [198]. Oral administration bioavailability is approximately 20% [207] with a concentration peak in 20–30 min [208]. Bioavailability of intrarectal administration is approximately 25% and intranasal is about 50% [200].

Limitations of pharmacokinetic data can limit the interpretation of dosing comparisons. As stated, the pharmacokinetics of norketamine are not well understood. While KET bioavailabilty after IV administration is high, it is also eliminated rapidly. However, because norketamine accumulates once a level of KET is reached, there is less need for more frequent KET. A dosing regimen of KET twice per day, or bolus doses, may keep KET levels high enough to allow the accumulation of norketamine without the need for IV administration.

### 4.5. Anti-Depressant Effects and Sex Differences

Here, we note that some dosing regimens produce anti-depressant-like effects with psychosis-like effects produced in others. Anti-depressant mechanisms may confound or interact with psychosis-like effects. These potential interactions may be a result of shared pathways involving synaptic plasticity, the glutamatergic system, and dopamine signaling [209,210]. Further, depression and psychosis are known to both involve abnormal synaptic plasticity [209,210]. Depressant drugs, like KET, block NMDA receptors, which induces anti-depressant effects and psychosis-like symptoms in healthy individuals [209]. In the glutamatergic system, the “glutamate burst” is associated with depressant action. A surge in extrasynaptic glutamate, followed by AMPA receptor activation seen in anti-depressants like KET, appears to be a key mechanism of the therapeutic effect of anti-depressants [210]. In psychosis, disruptions in glutamate signaling are central to the pathophysiology. Disruptions in the glutamate pathway could exacerbate psychosis [210].

The dopaminergic system is also affected by changes in glutamate [210] induced by depressants, and such changes can indirectly modulate dopamine release causing psychosis-like symptoms in some individuals [210].

It is of interest to note that both Manooki et al. [133] and Thelen et al. [124] observed sex differences in the expression of depressive-like behaviors in rats and mice, respectively. However, the effect of sub-chronic KET in the forced swim test was species-specific: the male mice exhibited anti-depressant-like effects, with reduced immobility in the forced swim test, while female mice displayed increased depressive-like behavior, with greater immobility. In contrast, the female Wistar rats exhibited reduced immobility compared to the male rats; that is, the females spent more time struggling, trying to escape, and less time immobile than the control rats [133]. Females also displayed behavior indicating heightened anxiety compared to males. This difference in affective behavior in females may be due to an increased sensitivity to the disruption of the glutamate pathway or possibly due to the higher clearance rate of KET in females compared to males.

## 5. Conclusions

This review highlights the robust utility of KET as a pharmacological model for psychosis-related symptoms in rodents. Both acute and sub-chronic IP administration of KET can induce a spectrum of positive, negative, and cognitive symptoms consistent with schizophrenia and bipolar disorder. Thus, the KET model offers considerable value for research. Through a comparison of a curated selection of 28 peer-reviewed empirical studies, his review offers three key conclusions. First, low doses administered over longer durations and high doses over shorter durations can produce equivalent long-term impairments if dosing is paired with appropriate washout periods. Second, behavioral impairments (e.g., in attention, memory, and affect) are reproducible and correlate strongly with structural, neurochemical, and electrophysiological alterations that parallel findings in human patients. Third, frequent dosing may serve as a proxy for maintaining steady KET levels with the need to resort to IV administration, enhancing the feasibility and translational relevance of rodent studies. This review supplies guidance to researchers of the benefits of various dosages, methods of administration, and timing of washout periods, to best match their research objectives. This knowledge may enable researchers to produce specific results more rapidly and assist with a transition to clinical trials. Ultimately, this synthesis supports KET as a flexible and validated tool in modeling psychiatric disorders. The findings serve as a practical guide for designing future studies based on specific cognitive or affective endpoints.

## Figures and Tables

**Table 1:** Summary of Behavioral and Neurological Effects of Varying Ketamine Dosing Schedules.

Species and Strain.	Ketamine Dose Regimen	Post-Injection Delay	Behavioral Task	Result	Summary of Findings	Neuro- Analysis	Structure Tested	Result	Summary of Findings	Reference
Rats Wistar	Acute 4, 8, 12, and 16 mg/kg SC	None	Open Field (motor activ.) Pre-Pulse Inhibition (attention) Discrete paired-trial T-maze (working memory)	Sig Sig Sig	Increased after 12 and 16 mg/kg Impaired after 4, 8 and 12 mg/kg Correct responses reduced after 12 mg/kg	c-Fos	DG, PFC, RSC, EC, HC, STR, NAc AMY, DMN	Sig	# of c-Fos-positive neurons increased in all regions, except STR	Imre, et al., 2006a [118]
Rats Wistar	Acute 4 and 12 mg/kg SC	None	Open Field (motor activ.) Pre-Pulse Inhibition (attention)	Sig Sig	Increased after 12 mg/kg Impaired after 12 mg/kg	HPLC NT level: GLU, DA, 5-HT, NA	DG	Sig	Decrease in GLU and DA No diff in 5-HT and NA	Imre et al., 2006b [119]
Rats Wistar	Acute 25 mg/kg IP	None	Open Field (motor activ.) LI (eyeblink) (learning, attention)	Sig Sig	Increased Impaired inhibition to pre-exp CS	In-vivo field potentials HPLC: GLU level	PFC-NAc NAc	Sig Sig	Increased amplitude Increased	Razoux et al., 2007 [120]
Rats Wistar	Acute 25 mg/kg IP racemic KET and S-KET	None	Open Field (motor activ.) 50 kHz USVs	Sig Not sig	Increased after KET and S-KET No diff in # of USVs	None				Wendler et al., 2016 [121]
Rats Wistar	Sub-chronic 25 mg/kg IP 1/day for 7 days; acute dose on day 8	None	Open Field (motor activ.)	Sig	Increased	Oxidative stress markers: TBARS, carbonyls, SH, CAT, SOD	PFC HC	Sig Sig	Increased TBARS, carbonyl Decreased CAT, SOD, SH (PFC)	Gazal et al., 2015 [122]
Rats Sprague Dawley	Sub-chronic 25 mg/kg IP 1/day for 7 days; acute dose on day 8	30 min	Open Field (motor activ.)	Sig	Increased	Oxidative stress markers: TBARS, SOD, CAT	HC PFC	No sig	No diff in any stress marker	Arslan et al., 2016 [123]
Rats Wistar	Sub-chronic 25 mg/kg IP 1/day for 14 days; acute dose on day 15	30 min	Open Field (motor activ.)	Sig	Increased	TBARS, carbonyls	PFC, HC, STR, AMY	Sig	TBARS and carbonyls increased in all regions	Ghedim et al., 2012 [124]
Rats Lister	Sub-chronic 10 and 30 mg/kg IP 1/day for 5 days	2 d	NMTS/OST (working memory)	Sig	Impaired after both doses up to 21 days post-KET	None				Rushforth et al., 2011 [125]
Rats Sprague–Dawley	Sub-chronic 30 mg/kg IP 1/day for 5 or 10 days	14 d	ASST (attention)	Sig	Impaired after 10 (but not 5) days of repeated KET	None				Nikiforuk & Popik 2012 [126]
Rats Long Evans	Sub-chronic 30 mg/kg IP 1/day for 5 days or 2x/day for 7 days	10 d	Associative Mismatch (attention) Object and Location Novelty Detection Delayed Match-to-Place T-Maze Elevated Plus Maze Neophagia (anxiety)	Sig Sig Sig Sig Not sig	Mismatch detection: Enhanced after 5-day tx; Impaired after 7-day tx Detection increased after 5-day tx Correct responses reduced after 7 d tx Less time in closed arms after 7-day tx No diff	None				Schumacher et al., 2016 [93]
Rats Long Evans	Sub-chronic 30 mg/kg IP 2x/day for 5 or 10 days	10 d	Delayed Spatial Win-Shift Radial-Arm Maze (working memory) Non-Delayed Random Foraging Task (working memory)	Sig Not sig	Errors and trials to criterion increased after 10 (but not 5) day tx Not impaired	None				Enomoto & Floresco 2009 [127]
Rats Sprague–Dawley	Chronic 30 and 80 mg/kg IP 1/day for 1, 2, and 3 mos *to freely moving rats*	None	None			EEG	NC, DH, AMY		Abnormal spike, hyper-sync bursting after both doses	Manohar et al. 1972 [128]
Rats Sprague–Dawley	2 and 5 mg/kg IV bolus; or 5, 10, or 20 mg/kg/h IV infusion	None	Open Field (motor activ.) Acoustic Startle (attention) Pre-Pulse Inhibition (attention) Hotplate Latency (thermal nociception)	Sig Not sig Sig Sig Sig	Increased after acute 2 and 5 mg/kg bolus; and 20 mg/kg/h; decreased after 5 mg/kg/h No diff after bolus tx; Reduced after 10 and 20 mg/kg/h Reduced after 5 mg/kg bolus and after 20 mg/kg/h Increased after 5 mg/kg bolus and after 10 and 20 mg/kg/h	None				Radford et al., 2017 [129]
Rats Wistar	0.25, 0.5, 1, 2, and 5 mg/kg, IV to rats under chloral hydrate-anesthesia	None	None			Single-unit activity Local field potentials	RtN, MD/CM thalamic nuclei, layer VI of the mPFC	Sig Sig Sig	Decreased firing rate of RtN, and MD/CM neurons after 1, 2, and 5 mg/kg; decreased firing rate of mPFC neurons after 2 and 5 mg/kg Decreased delta power (0.15–4 Hz) in RtN, mPFC after 1, 2, and 5 mg/kg; in MD/CM after 5 mg/kg Increased gamma power (30–90 Hz) in MD/CM. mPFC after 1, 2, and 5 mg/kg	Amat-Foraster et al., 2018 [130]
Rats Long–Evans	20 and 50 mg/kg IP to freely moving rats	None	None			Single-unit activity Local field potentials	Nucleus reuniens of thalamus CA1	Sig Sig Sig	Increased firing rate of reuniens and CA1 neurons after 50 mg/kg Delta power: increased in reuniens and CA1 after 50 mg/kg; no diff after 20 mg/kg Gamma power: Increased in reuniens and CA1 after 20 and 50 mg/kg	Zhang et al., 2012 [131]
Rats Sprague–Dawley	25 or 50 mg/kg IP to freely moving rats	None	None			Local field potentials	OlfC, OFC, PFC, STR, SepA, IntTh, SenC, TemAA, SenTh	Sig Sig	Increased mean LFP power spectrum across structures Decrease in functional connectivity, within and between brain regions	Nasretdinov et al., 2023 [132]
Rats Wistar	30 mg/kg IP 1x/day for 5 days	None	Open Field (motor activ.) Hotplate Latency (thermal nociception) Marble Burying (anxiety-related) Forced Swim Test (anti-depressant assay) NOR (long-term memory)	Sig Sig Not sig Sig Sig	Increased distance traveled in both sexes, but ♀ > ♂ Latency: decreased in ♀; no diff in ♂ # marbles buried: no diff Immobility time: reduced in ♀; no diff in ♂ Both sexes spent more time exploring familiar object	Real-time PCR (expression of BDNF mRNA)	PFC	Sig	Reduced expression in both sexes	Manooki et al., 2025 [133]
Mice C57BL/6J and 129SvPasIco	Acute 100 mg/kg IP	None	Pre-Pulse Inhibition (attention)	Sig	Reduced in both strains	None				Brody et al., 2003 [134]
Mice CD1 and C57BL/6JOlaHsd	Acute 10 and 20 mg/kg IP	15 min	5-CSRTT (attention)	Sig Sig	Premature responses: Increased after 10 mg/kg; Perseverative responses: increased after 10 and 20 mg/kg in C57	None				Oliver et al., 2009 [135]
Mice Swiss–Kunming	Acute and sub-chronic 25, 50, and 100 mg/kg IP 1/day for 7 days, acute dose on day 8	None	Open Field (motor activ.) Forced Swim Test (anti-depressant assay) Y-Maze (working memory) NOR (long-term memory)	Sig Sig Sig Not sig	Increased at all acute and sub-chronic doses Increased immobility after sub-chronic 100 mg/kg Decreased alternation after acute and sub-chronic 100 mg/kg No diff after any dose	Neuron count (NeuN-pos. cells) Oxidative stress markers: SOD, NO, MDA, NOS	HC PFC	Sig Sig Sig	After sub-chronic 100 mg/kg: HC: Reduced Reduced SOD, Increased NO, and NOS PFC: Increased NO and NOS	Hou et al., 2013 [136]
Mice C57BL/6J	Acute 20 mg/kg IP and sub-chronic 20 mg/kg IP 1/day for 7 days	1 h w/acute 7 days w/sub-chronic	Open Field (motor activ.) ASST (attention)	Not sig Sig	No diff after acute or sub-chronic Impaired after acute and sub- chronic	None				Szlachta et al., 2017 [137]
Mice C57BL/6N	Sub-chronic 30 mg/g IP 1/day for 14 days	24 h	Open Field (motor activ.) NOR (long-term memory)	Sig Sig	Reduced rearing Less time spent exploring novel obj.	PV-positive cells c-Fos	PFC, DH CA1 HC SUB, EC	Sig Sig	Reduced in both regions Reduced in CA1 and SUB; no diff in EC	Hauser et al., 2017 [97]
Mice C57BL/6J	Sub-chronic 30 mg/g IP 1/day for 5 days	5–7 days	Open Field (motor activ.) Sociability test Social Memory Y-Maze (spatial memory) Forced Swim Test (despair/anti-depressant-like assay) Tail Suspension Test (despair/anti-depressant-like assay) Acoustic Startle (attention) Pre-Pulse Inhibition (attention)	Sig Sig Sig Sig Sig Sig Sig Sig	Less time in center Decreased time in social interaction Impaired social recognition Reduced time in novel arm Immobility: increased Immobility: increased Increased startle response Reduced	None				Sultana & Lee (2020) [138]
Mice C57BL/6N	Sub-chronic 30 mg/g IP 1/day for 5 days	5 days	Open Field (motor activ.) Sociability test Social Memory test	Not sig Not sig Not sig	No diff from saline controls No diff from saline controls No diff from saline controls	None				Harda et al., 2022 [139]
Mice C57BL/6J	Sub-chronic 3, 5, or 10 mg/kg IP 1/day for 21 days	24 h	Open Field (motor activ.) Forced Swim Test (anti-depressant-like assay)	Sig Sig	Center exploration by ♀’s reduced after 5 and 10 mg/kg Immobility: decreased in ♂; increased in ♀ after 10 mg/kg	μg/g tissue: GLU, ASP, 5-HT, 5-HIAA, 5-HIAA/5-HT. Syntaxin-I, Synapsin-1, SNARE-100 kDa	HC, PFC	Sig Sig	HC: GLU, ASP: reduced in ♀; 5-HIAA, 5-HT turn-over increased in ♂; Synapsin-1, SNARE in-creased in ♂ PFC: Syntaxin reduced in ♀	Thelen et al. 2016 [140]
Mice C57BL/6J	Acute 30 mg/kg IP; and sub-chronic 30 mg/kg IP 2x/day for 7 days	7-day washout	NOR (long-term memory)	Sig	Reduced discrimination index after acute and sub-chronic	None				Rajagopal et al., 2016 [89]

**Abbreviations: Behavioral Tasks:** 5-CSRTT, five-choice serial reaction time test; ASST, attentional set-shifting task; LI, latent inhibition; NMTS/OST, non-match-to-sample/odor span task; NOR, object recognition memory task; TFC, trace fear conditioning. **Brain regions:** AMY, amygdala; DH, dorsal hippocampus; DMH, dorsomedial nucleus of hypothalamus; EC, entorhinal cortex; HC, hippocampus; IntTh, integrative nuclei of the thalamus; MD/CM, mediodorsal/centromedial nucleus of the thalamus; NC, neocortex; NAc, nucleus accumbens; OlfC, olfactory cortex; OFC, orbitofrontal cortex; PFC, prefrontal cortex; PVN, paraventricular nucleus of hypothalamus; RSC, retrosplenial cortex; RtN, reticular nucleus of thalamus; SenC, primary sensorimotor cortex; SenTh, sensory nuclei of the thalamus; SepA, septal area; STR, striatum; SUB, subiculum. **Neurochemical Tests and Measures:** 5-HIAA, 5-hydroxy-indoleacetic acid; 5-HT, serotonin (5-hydroxytryptamine); ASP, aspartate; CAT, catalase; GLU, glutamate; MDA, malondialdehyde; NO, nitric oxide; NOS, nitric oxide synthase; PV, parvalbumin; SH, sulfhydryl content; SOD, superoxide dismutase; SNARE, soluble *N*-ethylmaleimide-sensitive factor attachment protein receptor; and TBARS, thiobarbituric acid reactive substances. **Other:** #, number; tx, treatment; ♀, female; and ♂, male. **Note:** Index of the experimental details and results from the reviewed subset of published KET studies testing dose-dependent behavioral testing of schizophrenic-like symptoms and brain region involvement. Dose equivalents between mice and rats are approximately 2:1, respectively. Sig refers to the report of a significant effect of KET on the respective behavioral tasks or neurophysiological determinant, *p* < 0.05.

## Data Availability

No new data were created or analyzed in this study. Data sharing is not applicable to this article.

## Abbreviations

The following abbreviations are used in this manuscript:

5-CSRTT	5-choice serial reaction time test
KET	ketamine
NMDA	*N*-methyl-D-aspartate
5-HT	serotonin
